# Immunocyte senescence: A new perspective on the remodeling of the ovarian cancer microenvironment and therapeutic intervention

**DOI:** 10.1016/j.jpha.2025.101492

**Published:** 2025-11-07

**Authors:** Xiang Li, Xian Li, Sha Ni, Xiaohui Zhang, Bingnan Liu

**Affiliations:** a Department of Obstetrics and Gynecology, Shengjing Hospital of China Medical University, Shenyang, 110004, China; b Department of Ultrasound, Shengjing Hospital of China Medical University, Shenyang, 110004, China; c Department of Anesthesiology, Shengjing Hospital of China Medical University, Shenyang, 110004, China; d Department of Laboratory Medicine, Shengjing Hospital of China Medical University, Shenyang, 110004, China; e Liaoning Clinical Research Center for Laboratory Medicine, Shenyang, 110016, China

**Keywords:** Ovarian cancer, Immunocyte senescence, Tumor microenvironment, Therapeutic resistance, Targeted therapy

## Abstract

Immune cell senescence in ovarian cancer manifests as distinct phenotypic alterations in T cells, natural killer (NK) cells, macrophages, and dendritic cells (DCs), alongside dynamic crosstalk with the tumor microenvironment (TME). This senescence contributes substantially to the high mortality and therapeutic resistance characteristic of ovarian carcinoma. Senescent immune cells develop a senescence-associated secretory phenotype (SASP), secreting pro-tumorigenic cytokines and chemokines that drive regulatory T cell (Treg) expansion, extracellular matrix (ECM) remodeling, and the establishment of an immunosuppressive niche. Key drivers of immunocyte senescence include telomere erosion, epigenetic dysregulation, metabolic stress, DNA damage from chemotherapy, and chronic inflammatory signals. Emerging interventions, such as senolytic agents to selectively eliminate senescent immune cells, senomorphic compounds to attenuate SASP factors, and strategies to reprogram immune effectors, hold promise for restoring antitumor immunity and overcoming resistance to conventional therapies. A deeper understanding of the molecular mechanisms governing immunosenescence will be critical for the rational design of combination regimens and the development of next-generation immunotherapies in ovarian cancer.

## Highlights

• The senescence phenotypes of immunocyts are mapped for revealing their collective role in fostering an immune-evasive niche.
• Chemotherapy and chronic inflammation as exogenous accelerators of immunosenescence.
• Single-cell multi-omics and senescence-focused therapeutic windows are critical for personalized anti-TME strategies.

## Graphical abstract

**Figure:**
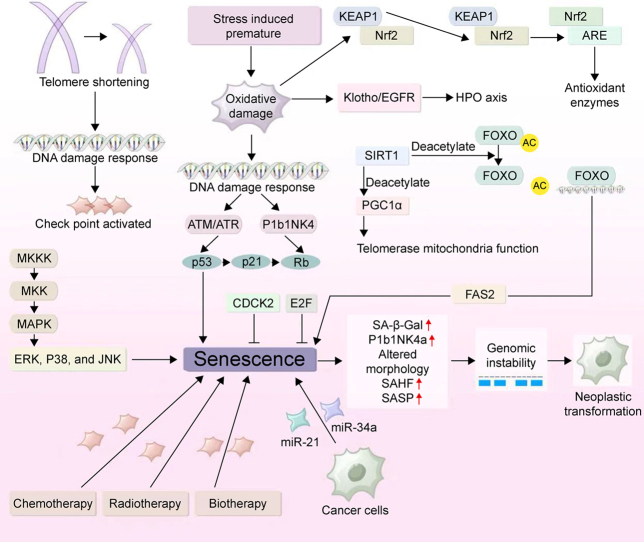

## 1 Introduction

Ovarian cancer ranks among the most lethal cancers affecting women, with a disheartening five-year survival rate of less than 50%. A staggering proportion of patients are diagnosed at advanced stages (III or IV), where therapeutic choices become severely constrained [1]. Even after achieving complete remission through aggressive surgical debulking and initial chemotherapy regimens, an alarming 70%–80% of patients experience relapse within 2–5 years. Ultimately, many succumb to the relentless chemotherapy resistance that characterizes recurrent ovarian cancer [1]. Chemotherapy resistance is closely intertwined with the immunosuppressive conditions prevalent within the tumor microenvironment (TME). Cells such as tumor-associated macrophages (TAMs) and fibroblasts contribute to this immunosuppressive landscape by secreting pro-inflammatory molecules like interleukin-6 (IL-6) and inhibitory cytokines such as transforming growth factor-beta (TGF-β). This environment facilitates tumor immune evasion, making it challenging for the immune system to effectively combat cancer growth [2]. Beyond general TME remodeling, ovarian carcinomas are genetically heterogeneous. High-grade serous tumors almost invariably harbor tumor protein p53 (TP53) mutations, whereas germline or somatic loss of breast cancer susceptibility gene 1/2 (*BRCA1/2*) induces homologous-recombination (HR) deficiency (HRD) with attendant neoantigen generation. Other subtypes feature phosphatidylinositol-4,5-bisphosphate 3-kinase catalytic subunit alpha (PIK3CA) pathway activation (clear cell/mucinous) or cyclin E1 (CCNE1) amplification (HR-proficient high-grade). These driver mutations critically modulate immune features: *BRCA*-mutant, HR-deficient tumors exhibit elevated tumor mutational burden, type I interferon (IFN) signaling via cyclic guanosine monophosphate-adenosine monophosphate (GMP-AMP) synthase (cGAS)-stimulator of IFN genes (STING), increased CD8^+^ T-cell infiltration and higher programmed death-ligand 1 (PD-L1) expression, whereas TP53-mutant tumors often recruit immunosuppressive M2 macrophages and regulatory T cells (Tregs). A deeper analysis of driver-specific TME programs, particularly BRCA-dependent crosstalk, is thus essential to complete the authors’ arguments.

In the TME, a complex ecosystem of cancer cells, stromal elements, extracellular matrix (ECM), vasculature, immune cells, and macrophages comprise over one-third of the immune cells and display remarkable plasticity, shifting between pro-inflammatory M1-like and anti-inflammatory M2-like states in response to local cues. During early tumorigenesis, M1 macrophages secrete type 1 helper T Cell (Th1)-associated cytokines (e.g., tumor necrosis factor alpha (TNF-α), IL-1β, IL-6, and IL-12) to mediate immunosurveillance and malignant cell clearance, but as tumors evolve, cancer-derived factors such as IL-4, IL-10, TGF-β, and colony-stimulating factor 1 (CSF-1) drive M2 polarization, fostering an immunosuppressive stroma that promotes angiogenesis, ECM remodeling, and metastatic dissemination. This M2-dominated niche is further reinforced by non-immune components, cancer-associated fibroblasts (CAFs), and a dense ECM, which hinders cytotoxic T cell infiltration and sustain chronic inflammation, thereby exacerbating tumor progression and resistance to therapy. Consequently, strategies aimed at reprogramming macrophages to reestablish a pro-inflammatory TME hold significant promise for restoring anti-tumor immunity and improving clinical outcomes [2].

However, the TME is highly plastic, and recent efforts aim to “reprogram” it to restore antitumor immunity and sensitize tumors to therapy. Strategies under investigation include blockade of pro-tumor cytokines (IL-6 and TGF-β) while boosting anti-tumor signals (IL-27), engineering the ECM and extracellular vesicles for targeted delivery of small interfering RNA (siRNAs) or small-molecule drugs, and combining immune checkpoint inhibitors with TGF-β inhibitors, radiotherapy, or oncolytic viruses to “soften” the stroma and enhance T cell activity. Optimized adoptive cell therapies (chimeric antigen receptor T cell (CAR-T) and tumor-infiltrating lymphocyte (TIL)) further benefit from microenvironmental pre-conditioning or genetic modifications that improve tumor homing and persistence. Looking forward, integrating single-cell and spatial transcriptomics will map the distribution and interactions of CAFs, TAMs, and TILs to reveal novel targets, while liquid-biopsy monitoring (exosomes and circulating tumor DNA (ctDNA)) and exploration of microbiome- or metabolism-based interventions may achieve dynamic control over TME remodeling. Together, these emerging approaches offer a roadmap to overcome ovarian cancer's innate resistance by transforming its microenvironment into one that favors immune clearance and durable responses [3].

Cellular senescence, a state of permanent cell cycle arrest, frequently occurs in response to various stressors, including DNA damage, telomere erosion, and metabolic distress [3]. Senescent cells produce a range of inflammatory cytokines and growth factors, collectively known as the senescence-associated secretory phenotype (SASP), which can modulate the behavior of adjacent cells [4]. In the context of ovarian cancer, cellular senescence influences tumor progression through diverse mechanisms [5]. For example, senescent cells stimulate the expansion of tumor progenitor cells by secreting osteonectin [6]. Furthermore, senescence in ovarian cancer cells is linked to chemotherapy-induced drug resistance. Cellular senescence exhibits a dual nature in cancer, serving both as function as an anticancer defense by permanently halting cell proliferation, thereby inhibiting tumor growth [7]. For example, chemotherapy-induced senescent cells (therapy-induced senescence (TIS)) activate antitumor immune responses by secreting SASP factors such as IL-6 and IL-8 [4,8]. On the other hand, senescent cells may also promote tumor progression by remodeling the TME. In ovarian cancer, for instance, senescent cells induce the generation of cancer stem cells (CSCs) through mechanisms such as upregulating nicotinamide phosphoribosyltransferase (NAMPT) and enhance cisplatin resistance [9]. Furthermore, senescent cells may support tumor growth by promoting angiogenesis and inflammatory responses [2,10].

Immunosenescence denotes the progressive deterioration of immune system efficacy over time. This process is closely linked to tumor immune evasion. For example, as age increases, T cell function declines, and antigen presentation capabilities weaken, resulting in reduced efficacy of immune checkpoint inhibitors (e.g., programmed cell death protein 1 (PD-1)/PD-L1 and cytotoxic T-lymphocyte-associated protein 4 (CTLA-4)) [[11], [12], [13]]. Furthermore, immunosenescence aids in the establishment of persistent inflammation and an immunosuppressive microenvironment, which in turn fuels tumor development and progression. For instance, immunosenescence-associated macrophages and dendritic cells (DCs) may inhibit antitumor immune responses by secreting pro-inflammatory or inhibitory factors [12,14].

Importantly, CAFs serve as master regulators of ECM remodeling within the ovarian TME. Originating from resident fibroblasts under the influence of tumor-derived cues (notably TGF-β), CAFs exhibit remarkable plasticity and encompass distinct subtypes, myofibroblast-like CAFs (myCAF), inflammatory CAFs (iCAF), antigen-presenting CAFs (apCAF), and ovarian-specific S1–S4 populations defined by markers such as alpha-smooth muscle actin (α-SMA), fibroblast activation protein (FAP), podoplanin (PDPN), and microfibril-associated protein 5 (MFAP5). Through bidirectional crosstalk with ovarian cancer cells, these CAF phenotypes activate signaling cascades that drive proliferation, invasion, and immune suppression. Concurrently, by secreting cytokines (e.g., IL-6 and IL-1β), chemokines, and matrix proteins (collagen, fibronectin, tenascin C), CAFs establish a reactive, drug-impermeable stroma that fosters metastasis, stem-like phenotypes, and chemoresistance. To interrogate these complex interactions in a physiologically relevant setting, recent work has introduced three-dimensional (3D) spheroid co-culture models, combining ovarian cancer cells with fibroblasts preconditioned by cancer cell-derived media, which faithfully recapitulate stromal-tumor architecture and dynamics, offering a robust platform to dissect CAF heterogeneity and to identify novel stromal targets for overcoming therapy resistance [12,14].

The ovarian adenocarcinoma TME is marked by severe hypoxia, aberrant vascularization, and extracellular acidification, each critically influencing senescence, senolysis, and immune regulation. Under hypoxic conditions, stabilization of hypoxia-inducible factor-1α (HIF-1α) in stromal and immune compartments drives the transcription of SASP factors (e.g., IL-6 and vascular endothelial growth factor (VEGF)) and enhances cyclin-dependent kinase inhibitor 1A (CDKN1A (p21)) and CDKN2A (p16) expression, promoting immunocyte senescence. Hypoxia also upregulates PD-L1 on both tumor and myeloid cells, dampening T-cell and NK-cell cytotoxicity, and induces anti-apoptotic B-cell lymphoma 2 (Bcl-2) family proteins that impair senolytic efficacy [12,14]. Tumor vascularization in ovarian cancer is characterized by VEGF-A-driven, tortuous, and leaky vessels that fail to normalize oxygen delivery. Endothelial cells within these dysfunctional capillaries acquire senescence-like phenotypes, exacerbate barrier breakdown, and limit both immune cell infiltration and uniform distribution of senolytic agents [12,14]. Metabolic reprogramming toward high glycolysis elevates lactate secretion, acidifying the extracellular milieu (pH 6.5, approximately). Acidification attenuates T-cell receptor signaling, skews macrophages toward an M2 phenotype, fosters Treg expansion and reduces effector lymphocyte cytotoxicity. Moreover, low pH hinders uptake and activity of small-molecule senolytics, suggesting that pH modulation could potentiate senolysis and restore antitumor immunity [12,14].

This paper will review the driving mechanisms of immunocyte senescence in ovarian cancer, the bidirectional regulation between senescent immunocytes and the TME, and the research progress on whether targeting immunosenescence can break through existing therapeutic bottlenecks. Building on this framework, in Section 2, we first delineate the key phenotypic hallmarks of senescent T cells, NK cells, macrophages and DCs within the ovarian TME, setting the stage for a functional analysis of their secretory and immunomodulatory roles. In this review, the term “ovarian cancer” refers to human ovarian adenocarcinomas.

## 2 Phenotypic features of senescent T cells, NK cells, macrophages, and DCs in the ovarian cancer microenvironment

### 2.1 Phenotypic hallmarks of senescent T cells, NK cells, macrophages, and DCs

#### 2.1.1 T cells: exhaustion and senescence (PD-1^+^/CD28^−^) and dysfunction

T cells exhibit characteristics of exhausted senescence and dysfunction in ovarian cancer. Exhausted senescence is manifested by high PD-1 expression, low CD28 expression, and metabolic reprogramming, leading to impaired T cell function and inability to effectively clear tumor cells [15,16]. Moreover, T cell senescence is linked to replicative senescence, a condition marked by the cessation of the cell cycle, heightened apoptosis, and a diminished immune response [15,17].

**Fig. 1:**
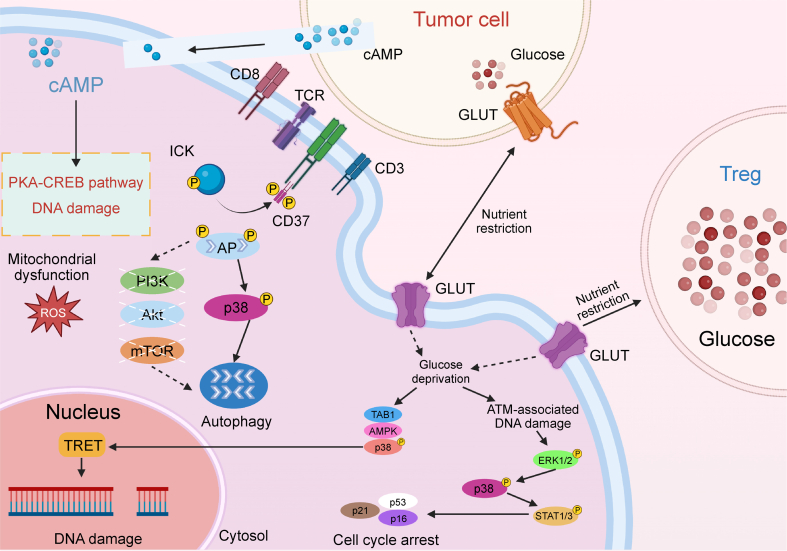
Underlying molecular pathways and signaling sequences driving T cell senescence within the tumor microenvironment (TME). Tumor-derived cyclic adenosine monophosphate (cAMP) triggers mitochondrial dysfunction and DNA damage in CD8^+^ T cells via protein kinase A (PKA)-cAMP response element-binding protein (CREB) signaling, while T-cell receptor (TCR) activation and nutrient deprivation engage phosphoinositide 3-kinase (PI3K)/protein kinase B (Akt)/mechanistic target of rapamycin (mTOR) and ataxia telangiectasia mutated (ATM)/p38 pathways to drive cytoprotective autophagy and senescence. Targeting these pathways (e.g., inhibiting p38 or autophagy) may reverse T-cell dysfunction and enhance antitumor immunity in next-generation immunotherapies. TCR: T-cell receptor; ICK: interleukin-2 (IL-2)-inducible T-cell kinase; AP: adaptor protein; ROS: reactive oxygen species; TRET: telomerase reverse transcriptase; GLUT: glucose transporter; TAB1: TAK1-binding protein 1; AMPK: AMP-activated protein kinase; ATM: ataxia telangiectasia mutated; ERK1/2: extracellular signal-regulated kinase 1/2; STAT1/3: signal transducer and activator of transcription 1/3.

The process of exhausted and senescent T cell development is complex and involves multiple mechanisms. At the molecular level, telomeres gradually shorten, and as the number of cell divisions increases, cells enter a senescent state and are unable to continue dividing once telomeres reach a certain length [18]. Concurrently, the expression of CD28 on the surface of T cells declines, leading to impaired function [19]. Furthermore, T cells become arrested at a certain stage of the cell cycle during senescence, unable to progress to the next division cycle [20]. When it comes to signal transduction, T cells found in chronic infections and within the TME express an array of inhibitory receptors-including PD-1, CTLA-4, and T-cell immunoglobulin, and mucin-domain containing protein 3 (TIM-3) and T-cell immunoreceptor with Ig and ITIM domains (TIGIT), that upon ligand engagement, suppress T cell receptor (TCR) signaling and impair effector function. By binding to their respective ligands, these receptors serve to suppress T cell activation and impair their functionality [19,21]. TCR signaling is also weakened, resulting in decreased T cell function [21]. Senescent T cells also alter their metabolic pathways, leading to energy deficiency and subsequent dysfunction [22]. Functionally, senescent T cells exhibit decreased proliferation, making them ineffective against infections or tumors [23]; reduced cytokine secretion, failing to produce sufficient factors crucial for immune responses such as IFN-γ and TNF-α [18]; and diminished killing ability, unable to effectively clear pathogens or tumor cells [23]. Other mechanisms include DNA damage and repair deficiencies, where senescent T cells often exhibit decreased DNA damage repair capacity [[24], [25], [26]]; mitochondrial dysfunction, leading to insufficient cellular energy supply and further impairing T cell function [27]; and epigenetic changes, such as alterations in DNA methylation and histone modifications, which also affect T cell function [15]. In terms of immune checkpoints, increased expression of PD-1 and other immune checkpoints is a significant marker of T cell exhaustion, leading to decreased T cell function. Blocking PD-1 can restore T cell function, providing a new strategy for treatment [21,23,28]. Age-related factors also influence T cell exhausted senescence, as T cells gradually enter a senescent state and lose function with age. This natural aging process is associated with chronic inflammation and oxidative stress, which accelerate T cell senescence and lead to immune dysfunction [29]. Table 1 provides a concise comparison between exhausted T cells and senescent T cells. Meanwhile, Fig. 1 offers a visual representation of the intricate molecular mechanisms and signaling cascades that govern T cell senescence within the TME.

**Table 1:** Distinctive features of exhausted and senescent T cells.

Characteristic	Exhausted T cells	Senescent T cells
Growth and cell cycle	Proliferation is compromised; cell cycle is halted (marked by elevated p27 and p15, and reduced cyclin E-CDK2 and CDC25A)	Proliferation is impaired; cell cycle is arrested (characterized by increased p16, p21, and p53 levels); DDR is activated; telomeres shorten and telomerase activity diminishes; SA-β-Gal activity rises
Triggering factors	Continuous exposure to antigens	Repeated exposure to stimuli; presence of DNA-damaging agents; exposure to stress signals
Surface marker	Elevated expression of PD-1, CTLA-4, TIM-3, LAG-3, BTLA, TIGIT, CD244, CD160, etc.	Absence of CD27 and CD28; high expression of TIM-3, CD57, CD45RA, TIGIT, KLRG-1, etc.
Functional shifts	Impaired cytotoxic function and decreased effector molecule production	Decreased cytotoxic capacity and decreased production of effector proteins; augmented regulatory activities.
TCR signaling pathway	Reduced activity of Lck and ZAP70	Reduced activity of the Lck/ZAP70/DLG1/Lat/SLP-76 signaling cascade
Cytokine profile	Early phase: IL-2 reduction	Elevated SASP; increased pro-inflammatory cytokines (e.g., IL-2, IL-6, IL-8, TNF, IFN-γ) and inhibitory cytokines (IL-10 and TGF-β)
	Mid-phase: TNF reduction	
	Late phase: IFN-γ and B-chemokines reduction	
Transeriptional profling	Increased levels of transcription factors: NFAT, NR4A, TOX, MYB, TCF-1, etc.	Increased levels of transcription factors: Foxp3, TFAM, etc.
	Progenitor exhausted T cells: high T-bet, low Eomes, and intermediate PD-1	
	Terminally exhausted T cells: low T-bet, high Eomes, and high PD-1	
Epigenetic changes	DNA methylation patterns associated with exhaustion	Increase in SAHF
Metabolic changes	Reduced glycolysis and mitochondrial biogenesis; accumulation of ROS	Increased glycolysis; reduced mitochondrial biogenesis; accumulation of ROS

CDK2: cyclin-dependent kinase 2; CDC25A: cell division cycle 25A; DDR: DNA damage response; SA-β-Gal: senescence-associated beta-galactosidase; PD-1: programmed cell death protein 1; CTLA-4: cytotoxic T-lymphocyte-associated protein 4; TIM-3: T-cell immunoglobulin and mucin-domain containing-3; LAG-3: lymphocyte-activation gene 3; BTLA: B and T lymphocyte attenuator; TIGIT: T-cell immunoreceptor with Ig and ITIM domains; KLRG-1: killer cell lectin-like receptor subfamily G member 1; Lck: lymphocyte-specific protein tyrosine kinase; ZAP70: zeta-chain-associated protein kinase 70; DLG1: discs large homolog 1; Lat: linker for activation of T cells; SLP-76: SH2 domain-containing leukocyte protein of 76 kDa; IL-2: interleukin-2; TNF: tumor necrosis factor; IFN-γ: interferon gamma; SASP: senescence-associated secretory phenotype; TGF-β: transforming growth factor beta; NFAT: nuclear factor of activated T-cells; NR4A: nuclear receptor subfamily 4 group A; TOX: thymocyte selection-associated high mobility group box protein; MYB: myeloblastosis oncogene; TCF-1: T-cell factor 1; T-bet: T-box transcription factor TBX21; Foxp3: forkhead box P3; TFAM: transcription factor A, mitochondrial; SAHF: senescence-associated heterochromatic foci; ROS: reactive oxygen species.

#### 2.1.2 Natural killer (NK) cells: diminished cytotoxicity and aberrant killer cell immunoglobulin-like receptor (KIR) expression

**Fig. 2:**
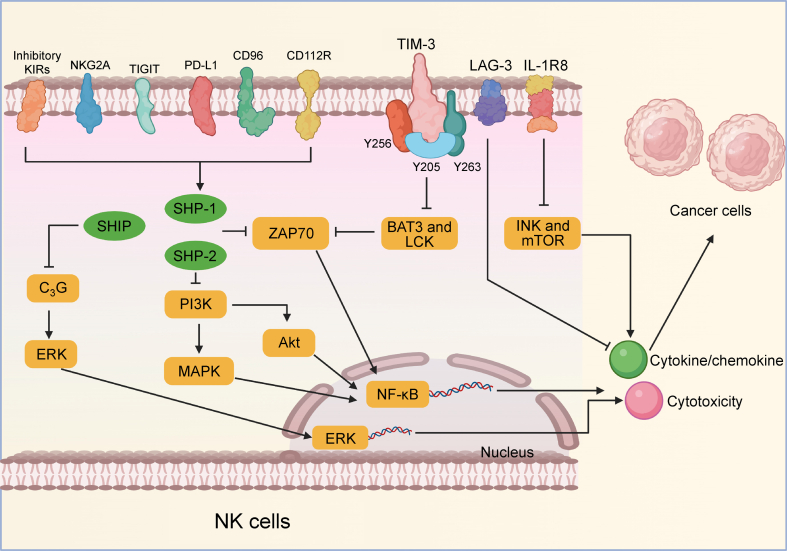
Natural killer (NK) cell inhibitory receptor signaling promotes tumor immune escape. Tumor cells express ligands (human leukocyte antigen class I (HLA-I), poliovirus receptor (PVR)/CD155, and programmed death-ligand 1 (PD-L1)) that engage NK inhibitory receptors (killer-cell immunoglobulin-like receptors (KIRs), NK group 2 member A (NKG2A), T-cell immunoreceptor with Ig and ITIM domains (TIGIT), and programmed cell death protein 1 (PD-1)), recruiting Src homology 2 domain-containing phosphatases (SHP-1/2) and SH2 domain-containing inositol phosphatase (SHIP) to suppress phosphoinositide 3-kinase/protein kinase B (PI3K/Akt), extracellular signal-regulated kinase (ERK), and nuclear factor kappaB (NF-κB) signaling, leading to NK cell dysfunction. Potential interventions include: dual receptor blockade (e.g., KIRs + TIGIT), SHP-1/2/SHIP inhibition, mammalian target of rapamycin (mTOR) activation, or cytokine therapy (interleukin-15 (IL-15)/IL-12) to restore NK cell cytotoxicity. →: leads to/activates; TIM-3: T-cell immunoglobulin and mucin-domain containing-3; LAG-3: lymphocyte-activation gene 3; IL-1R8: IL-1 receptor 8; C3G: Rap guanine nucleotide exchange factor 1; ERK: extracellular signal-regulated kinase; MAPK: mitogen-activated protein kinase; ZAP70: zeta-chain-associated protein kinase 70; BAT3: HLA-B-associated transcript 3; LCK: lymphocyte-specific protein tyrosine kinase; INK: inhibitor of nuclear factor kappa B kinase.

The aberrant expression of KIR in NK cells and its impact on tumor immune escape in ovarian cancer can be analyzed from multiple aspects. First, the causes of aberrant KIR expression include genetic polymorphism and environmental factors. KIR genes exhibit high polymorphism, resulting in significant differences in KIR gene expression among individuals. This polymorphism may affect NK cell function and its performance in ovarian cancer [30]. Additionally, environmental factors such as infections, inflammation, and drug exposure can also influence KIR gene expression and function. The TME also has a pivotal influence on the abnormal expression of KIRs. In the TME, inhibitory receptor signaling in NK cells is essential. Tumor cells evade NK cell attack by downregulating the expression of MHC-I molecules, leading to weakened inhibitory receptor (such as KIR) signaling in NK cells [31,32], thereby reducing NK cell killing activity and promoting tumor cell immune escape [31] (Fig. 2). Simultaneously, changes in KIR-human leukocyte antigen (HLA) pairing also affect NK cell function. KIR recognizes and kills cells that express low or no HLA ligands. In ovarian cancer, tumor cells may escape NK cell recognition and killing by altering the expression pattern of HLA molecules (such as downregulating HLA-E molecule expression) [33]. The engagement of immune checkpoints holds a significant position in the pathogenesis of ovarian cancer. Overactivation of suppressive receptors such as NK group 2 member A (NKG2A), KIR, and PD-1 weakens NK cell killing ability, leading to NK cell functional exhaustion and promoting tumor immune escape [34]. Furthermore, overactivation of inhibitory receptors may further promote tumor immune escape by modulating the role of alternative immune cells (including T cells and macrophages).

#### 2.1.3 Macrophages: M2 polarization tendency and metabolic reprogramming

Macrophages in ovarian cancer tend towards M2 polarization, accompanied by metabolic reprogramming. M2 macrophages exhibit immunosuppressive properties, promoting immune escape in the TME [35,36]. Additionally, macrophage senescence is characterized by decreased phagocytic activity and reduced inflammatory factor secretion [37,38]. The M2 polarization process of macrophages in ovarian cancer and its impact on immune escape constitute a complex and crucial mechanism. Studies have shown that macrophages can be reprogrammed into M2 macrophages through multiple mechanisms. These cells can secrete pro-inflammatory and pro-angiogenic factors, thereby promoting tumor growth and metastasis while inhibiting immune responses and helping tumor cells evade immune system recognition and clearance [39]. Furthermore, elevated heme-binding protein 2 (HEBP2) expression levels are significantly correlated with the half-maximal inhibitory concentration (IC_50_) of various chemotherapeutic drugs, suggesting that HEBP2 may promote multidrug resistance in ovarian cancer [40] and further assist ovarian cancer cells in evading immune system clearance by promoting macrophage polarization [39,41]. In terms of metabolic reprogramming, the metabolic state of macrophages is a critical aspect of their polarization process. Pro-inflammatory factors shift macrophages towards an M1-like phenotype, primarily utilize aerobic glycolysis and fatty acid synthesis under inflammatory conditions, whereas anti-inflammatory signals induce a shift towards an M2-like phenotype, which relies more heavily on oxidative phosphorylation (OXPHOS) and fatty acid oxidation for energy [[42], [43], [44]]. This metabolic reprogramming phenotype switch plays a vital role in macrophage polarization, but its specific mechanisms remain incompletely understood. The impact of macrophage M2 polarization on immune escape is mainly reflected in the following aspects: first, the pro-inflammatory and pro-angiogenic factors secreted by M2 macrophages fuel tumor expansion and the spread of cancer to other parts of the body; second, they inhibit immune responses, helping tumor cells evade immune system recognition and clearance; and finally, elevated HEBP2 expression levels are associated with multidrug resistance, further promoting tumor immune escape.

Moreover, ovarian cancer cells and their surrounding stroma overexpress CD47, which engages signal regulatory protein α (SIRPα) on macrophages to deliver a powerful “don't-eat-me” signal that blocks phagocytic clearance and thereby facilitates immune escape. At the same time, CD47 binds thrombospondin-1 (TSP-1), activating downstream protein kinase B/mechanistic target of rapamycin (Akt/mTOR) and nuclear factor-kappaB (NF-κB) pathways that not only enhance survival of tumor and stromal cells but also promote a senescence-like, dormant microenvironment by inhibiting angiogenesis and preventing removal of stressed or senescent cells. By both disabling macrophage clearance and enforcing a survival-dormancy axis, CD47 thus consolidates immunosuppression and therapeutic resistance within the ovarian TME [[42], [43], [44]].

Translational targeting of TAMs has now entered clinical evaluation. Imaging approaches such as gallium-68-labeled 1,4,7-triazacyclononane-1,4,7-triacetic acid (NOTA)-conjugated anti-macrophage mannose receptor variable domain of heavy chain of heavy-chainantibody 2 (VHH2) positron emission tomography/computed tomography (^68^Ga-NOTA-anti-MMR-VHH2 PET/CT) enable noninvasive visualization of M2-polarized macrophages in patients (e.g., Trail No.: NCT04168528) [45], while small-molecule inhibitors of the CSF-1-CSF-1 receptor (CSF-1-CSF-1R) axis (for example, ARRY-382 in Trail No.: NCT01316822) [46] are being tested to limit TAM survival and differentiation in solid tumors. Combination regimens, such as CSF-1R blockade plus pembrolizumab or pexidartinib, have demonstrated the ability to repolarize TAMs toward a pro-inflammatory, M1-like state, restoring cytotoxic immunity without ablating macrophage homeostatic functions. Cutting-edge single-cell and spatial transcriptomic profiling further refines our understanding of TAM heterogeneity, offering a path to selectively deplete or re-educate pro-tumor subsets and thereby sharpen patient stratification for macrophage-directed therapies [[42], [43], [44]].

#### 2.1.4 DCs: declining antigen presentation capability

DCs play a vital role as antigen-presenting cells (APCs) within the immune system, tasked with displaying tumor antigens to T cells and triggering immune responses. With age, the maturation and function of DCs are impacted in multiple ways, with these changes manifesting differently in various DC types. In myeloid DCs (mDCs), their number increases with age, but their maturity declines, evidenced by reduced exhibition of costimulatory markers (including CD86 and CD83) [47]. Additionally, the phagocytic capacity of mDCs in elderly individuals is significantly reduced, and their migratory ability is impaired, particularly in response to chemokines (such as macrophage inflammatory protein-3 beta (MIP-3β) and matrix metalloproteinase-3 (MMP-3)) [48,49]. In terms of cytokine secretion, elderly mDCs produce significantly lower levels of IL-12p40 and IL-6 under lipopolysaccharide (LPS) stimulation [50], and peripheral blood DCs (pDCs) produce significantly lower levels of IFN-α under polyI:C stimulation [51]. Elderly mDCs exhibit weakened ability to induce CD4^+^ and CD8^+^ T cells [52] and enhanced reactivity to self-antigens (such as DNA), characterized by elevated production of IFN-α and IL-6, which may be linked to the progression of chronic inflammation and autoimmune disorders [53]. During their maturation process, immature DCs display decreased expression of costimulatory molecules (like CD80 and CD86) and major histocompatibility complex class II (MHC-II) molecules, along with reduced secretion of pro-inflammatory cytokines (such as IL-12 and IFN-γ). These alterations result in compromised antigen presentation and diminished T cell activation abilities of the DCs [54,55].

In ovarian cancer, the declining antigen presentation capability of DCs involves multiple molecular mechanisms. First, the expression of MHC-II molecules on DCs is significantly downregulated, directly affecting their ability to present antigens to CD4^+^ T cells [56] and subsequently inhibiting T cell activation and function. Simultaneously, the expression of costimulatory molecules is also downregulated, playing a fundamental role in T cell activation. The reduced expression of these molecules further weakens the antigen presentation capability of DCs [57]. As individuals age, the ability of DCs to mature and present antigens diminishes, thereby weakening the immune system's capacity to identify and eliminate tumors [36,37]. The ovarian cancer microenvironment is abundant in immunosuppressive factors such as TGF-β and prostaglandin E2 (PGE2). These factors not only directly inhibit DC function but also exacerbate the immunosuppressive environment by affecting the activity of other immune cells (such as Tregs and macrophages). TGF-β and PGE2 can reduce the expression of MHC-II and costimulatory molecules on DCs, significantly impairing their antigen presentation capability [58]. Furthermore, DC differentiation and function in the ovarian cancer microenvironment are disrupted. Studies have shown that DCs may be converted into myeloid-derived suppressor cells (MDSCs) with immunosuppressive functions. These cells additionally suppress T cell function by releasing immunosuppressive molecules, including transforming growth TGF-β and PGE2. Meanwhile, the expression levels and recognizability of tumor-associated antigens (TAAs) expressed by tumor cells in the microenvironment are influenced by multiple factors. For instance, certain TAAs may be secreted into the microenvironment through mechanisms such as exosomes, interfering with DC antigen capture and presentation capabilities [59,60]. TAMs and tumor-associated neutrophils (TANs) inhibit DC maturation and antigen presentation capabilities by secreting factors such as IL-10 and TGF-β [61]. Additionally, the hypoxic state and metabolites (such as lactate) in the TME also impair DC function [62]. Finally, metabolic and signaling pathways in DCs are also abnormal in ovarian cancer. For example, the production of LPS-responsive outer membrane vesicles (ORMVs) increases, interfering with normal DC function and leading to decreased antigen presentation capability. These molecular mechanisms collectively contribute to the significant decline in DC antigen presentation capability in ovarian cancer, impairing the immune system's capacity to detect and eliminate tumors.

The diminished functionality of DCs not only hinders the immune system's ability to recognize and eliminate tumors but also has a direct bearing on the prognosis of ovarian cancer patients. Research indicates that lower levels of resting DCs in ovarian cancer patients correlate with reduced survival rates [63]. Moreover, DC function undergoes further deterioration in patients with advanced ovarian cancer, potentially due to the heightened levels of immunosuppressive factors present within the TME [64]. While Section 2.1 characterized how immunocyte senescence alters cellular phenotypes, the following Section 2.2 examines how the SASP actively remodels the ovarian TME through cytokine- and matrix-mediated immunomodulation.

### 2.2 Immunomodulatory functions of the SASP

Senescent cells orchestrate a complex cytokine milieu that can both restrain and fuel tumor progression. Pro-inflammatory SASP components, most notably IL-1β, IL-6, and IL-23, promote protumorigenic inflammation, drive epithelial-mesenchymal transition (EMT) and enhance stromal activation, whereas immunosuppressive factors such as TGF-β and IL-10 suppress effector lymphocytes and support metastatic dissemination. This dichotomy underscores the challenge of targeting a cytokine network that exhibits concentration-dependent, receptor-specific and temporally dynamic effects, and highlights key gaps in our understanding of how SASP kinetics and receptor heterogeneity dictate durable reprogramming of the ovarian TME [64].

Building on these mechanistic insights, several strategies have entered preclinical and clinical evaluation to reprogram the aged, immunosuppressive niche. Low-dose IL-2 or IL-2/anti-IL-2 complexes are employed to bolster NK and CD8^+^ T-cell cytotoxicity, while neutralizing antibodies against IL-6 or TGF-β aim to abrogate deleterious SASP loops. Anti-VEGF therapies (e.g., bevacizumab) further dismantle aberrant vasculature driven by senescent stroma. Cutting-edge approaches also seek to exploit extracellular vesicle–cytokine crosstalk for precision delivery of modulatory signals. Collectively, these interventions aspire to convert an “aged” TME, perpetuated by IL-6, IL-8, and TGF-β, into an immunostimulatory environment conducive to sustained antitumor immunity [64].

#### 2.2.1 IL6/IL8/TGFβ promote Treg cell expansion

In ovarian cancer, senescent stromal and tumor cells release a concerted SASP signature, chiefly IL-6, IL-8, and TGF-β, that initiates a sequential cascade driving Treg-mediated immunosuppression. i) Chemokine-driven recruitment: elevated IL-6 and IL-8 gradients attract C–C chemokine receptor type 4 (CCR4)^+^ and C–X–C chemokine receptor type 1/2 (CXCR1/2)^+^ Treg precursors from circulation into the tumor parenchyma [64]. ii) Local differentiation and proliferation: within the microenvironment, TGF-β engages mothers against decapentaplegic homolog 2/3 (SMAD2/3) signaling to convert naïve CD4^+^ T cells into forkhead box P3 (Foxp3)^+^ Tregs, while IL-6 activates signal transducer and activator of transcription 3 (STAT3) to support their IL-2-independent expansion [64]. iii) Suppressive reinforcement: expanded Tregs secrete high levels of IL-10 and additional TGF-β, which directly inhibit cytotoxic CD8^+^ T cell function, reducing perforin and granzyme production, and downregulate co-stimulatory molecules (CD80/CD86) on DCs, impairing antigen presentation [64]. iv) Retention and potency enhancement: concurrent SASP-induced upregulation of C–C motif chemokine ligand 22 (CCL22) in tumor and stromal cells promotes CCR4-mediated Treg retention, and TGF-β-driven p38/mitogen-activated protein kinase (MAPK) pathway activation further heightens their suppressive capacity [64]. v) Pathophysiological impact: the sustained accumulation of highly suppressive Tregs cooperates with MDSCs and pro-angiogenic macrophages to forge a resilient immunosuppressive niche, which correlates with aggressive tumor growth, chemoresistance, and poorer progression-free and overall survival in patients [64].

#### 2.2.2 MMPs mediate matrix remodeling and immune suppressive barrier formation

**Fig. 3:**
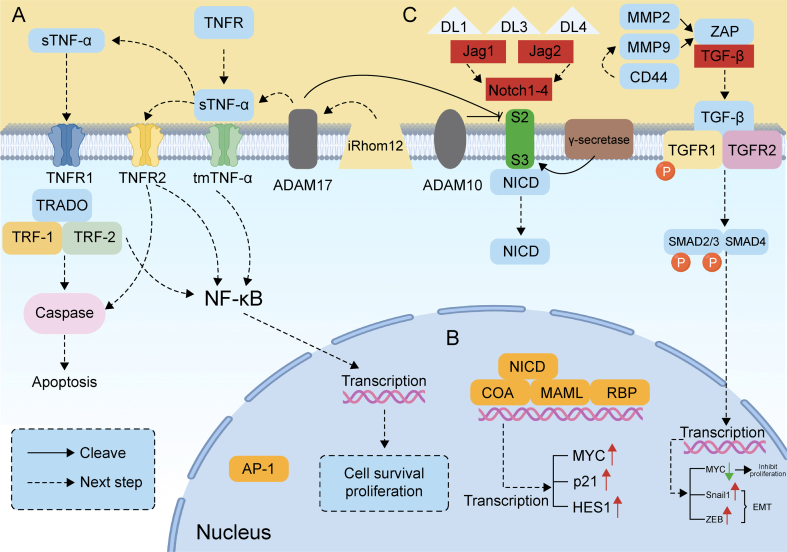
Immune-related matrix metalloproteinase (MMP) signaling in ovarian cancer. Tumor necrosis factor-alpha (TNF-α), Notch, and transforming growth factor-beta (TGF-β) pathways converge to regulate tumor behavior: (A) a disintegrin and metalloproteinase 17 (ADAM17)-cleaved TNF-α activates TNF receptor 1/2 (TNFR1/2), driving nuclear factor kappaB (NF-κB)-mediated survival or apoptosis; (B) Notch ligands induce ADAM10/γ-secretase cleavage, releasing Notch intracellular domain (NICD) to upregulate MYC and hairy and enhancer of split-1 (HES1); and (C) MMP2/9 and CD44 release TGF-β, which promotes epithelial-mesenchymal transition (EMT) via mothers against decapentaplegic homolog 2/3/4 (SMAD2/3/4). Targetable nodes include ADAM17/10, γ-secretase, MMPs, and SMAD signaling. sTNF-α: soluble TNF-α; TNFR: TNF receptor; tmTNF-α: transmembrane TNF-α; iRhom2: inactive rhomboid-like protein 2; TRAF1: TNF receptor-associated factor 1; AP-1: activator protein 1; COA: MAML: mastermind-like transcriptional coactivator; MYC: MYC proto-oncogene; HES1: hairy and enhancer of split-1; Snail1: snail family transcriptional repressor 1; ZEB: zinc finger E-box binding homeobox; DLL1: delta-like ligand 1; Jag1: jagged 1; Notch1-4: neurogenic locus notch homolog protein 1-4; ZAP: zeta-associated protein; TGFBR1: transforming growth factor-beta receptor 1.

MMPs promote the formation of immune suppressive barriers in the TME through multiple mechanisms. First, MMPs degrade collagen and other components in the ECM, promoting angiogenesis. This process provides necessary nutrients and oxygen to tumors while also providing channels for immune cell infiltration. However, newly formed blood vessels are often utilized by tumor cells to evade immune surveillance [65], thereby forming an immunosuppressive environment. Second, MMPs regulate immune cell function; for example, MMP-9 can inhibit T cell migration and activation, reducing antitumor immune responses [66]. Furthermore, MMPs promote the formation of an immunosuppressive environment by degrading immune-regulatory molecules in the ECM (such as laminin and elastin) [67]. MMPs also promote immune escape by degrading ECM components to facilitate tumor cell migration and invasion, helping tumor cells evade immune surveillance. For instance, MMP-9 can promote tumor cell entry into the bloodstream and metastasis to other sites [68]. Simultaneously, MMPs further promote immune escape by degrading receptors on the exterior of immune cells (e.g., CD44) [69,70]. Additionally, MMPs affect immune cell infiltration and distribution by degrading ECM components. For example, MMP-9 can inhibit the infiltration of TILs, reducing antitumor immune responses [69]. MMPs also promote the formation of immune suppressive barriers by activating inflammatory responses and promoting the recruitment and activation of immunosuppressive cells [71]. MMPs occupy a vital position in the TME, promoting the formation of immune suppressive barriers through mechanisms such as angiogenesis promotion, immune cell function regulation, immune escape facilitation, immune cell infiltration influence, and inflammatory response activation (Fig. 3).

#### 2.2.3 Regulatory association between SASP and PD-L1 expression

**Fig. 4:**
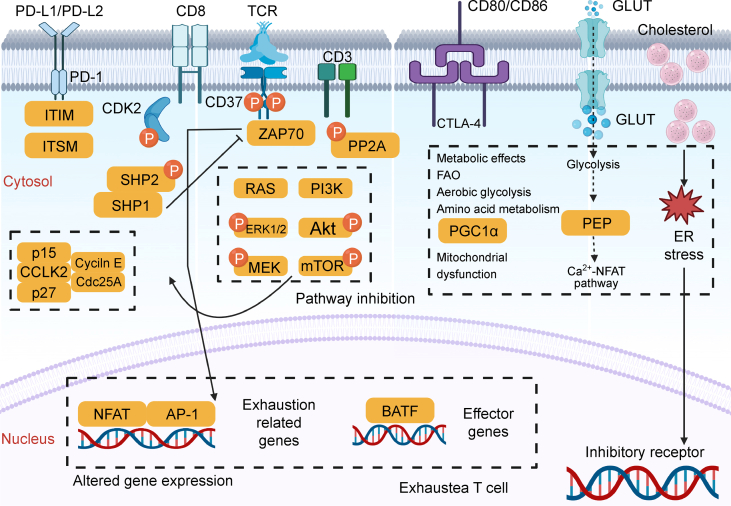
Programmed cell death protein 1 (PD-1) and cytotoxic T-lymphocyte-associated protein 4 (CTLA-4) signaling, along with metabolic modulation, inducing T cell exhaustion within the tumor microenvironment (TME). Programmed death protein 1 (PD-1) engagement by programmed death-ligand 1 (PD-L1)/PD-L2 recruits Src homology 2 domain-containing phosphatase 1 (SHP1)/SHP2 to suppress T cell receptor (TCR)-proximal (zeta-chain-associated protein kinase 70 (ZAP70)) and distal (rat sarcoma (RAS)-mitogen-activated protein kinase kinase (MEK)-extracellular signal-regulated kinase (ERK), phosphoinositide 3-kinase (PI3K)-protein kinase B (Akt)-mechanistic target of rapamycin (mTOR)) signaling, while CTLA-4-CD80/CD86 interactions impair metabolic fitness via peroxisome proliferator-activated receptor gamma coactivator 1-alpha (PGC1α)/phosphoenolpyruvate (PEP) downregulation, inducing mitochondrial/endoplasmic reticulum (ER) stress and calcium (Ca^2+^)-nuclear factor of activated T cells (NFAT) dysregulation. These convergent pathways establish an exhausted transcriptional state (elevated nuclear factor of activated T cells (NFAT)/activator protein 1 (AP-1), suppressed basic leucine zipper ATF-like transcription factor (BATF)) that can be targeted through phosphatase inhibition, metabolic modulation or transcription factor blockade to restore antitumor immunity. GLUT: glucose transporter; ITIM: immunoreceptor tyrosine-based inhibitory motif; ITSM: immunoreceptor tyrosine-based switch motif; CDK2: cyclin-dependent kinase 2; CCLK2: CDK-like 2; Cdc25A: cell division cycle 25A; PP2A: protein phosphatase 2A; FAO: fatty acid oxidation.

In ovarian cancer, particularly high-grade serous ovarian cancer, PD-L1 expression is elevated. This expression is tightly linked to the tumor immune microenvironment and may aid in tumors evading immune system detection by suppressing T cell activity [72,73]. The level of PD-L1 expression also correlates with the effectiveness of immunotherapy, as ovarian cancer patients with high PD-L1 expression tend to exhibit better responses to immune checkpoint inhibitors (e.g., pembrolizumab) [74]. SASP factors may regulate PD-L1 expression by influencing immune cell function, such as activating or inhibiting the activity of T cells and other immune cells, thereby indirectly affecting PD-L1 expression [75]. Although direct evidence is limited, it is speculated that SASP factors may influence PD-L1 expression by affecting immune cells in the TME. For example, SASP factors may increase PD-L1 expression by promoting the accumulation of immunosuppressive cells (such as Tregs) [75,76]. Additionally, SASP factors may indirectly regulate PD-L1 expression by affecting signaling pathways (such as the Janus kinase (JAK)/STAT signaling pathway) within tumor cells (Fig. 4). While the SASP-mediated upregulation of PD-L1 and T cell exhaustion has been documented in multiple carcinomas (e.g., lung, colorectal, and breast), ovarian adenocarcinomas exhibit unique peritoneal niche amplification of IL-6/IL-8 gradients within malignant ascites. This ascites-rich microenvironment, shaped by ovarian-specific CAF subsets (S1–S4), sustains prolonged STAT3 activation in CD8^+^ TILs, driving a more severe exhaustion phenotype than seen in solid-organ confined tumors. In contrast, lung cancers rely more heavily on MDSC-driven suppression and colorectal tumors on fibroblast-derived ECM stiffening. These site-specific differences underscore the need to target ascites-associated SASP networks in ovarian cancer.

CD39 shapes the immunosuppressive TME primarily through its ectonucleotidase activity: by hydrolyzing pro-inflammatory extracellular adenosine triphosphate (ATP) into AMP (and, via CD73, into adenosine), it generates high local adenosine concentrations that engage adenosine A_2A_ receptor (A_2A_R) and adenosine A_2B_ receptor (A_2B_R) receptors on effector immune cells. This signaling cascade potently inhibits T cell, NK cell, and DC functions while promoting the expansion and suppressive activity of Tregs and MDSCs, effectively recapitulating the paracrine immunosuppressive milieu driven by SASP factors such as IL-6, IL-8, and TGF-β. Beyond direct immune cell modulation, CD39 is highly expressed on stromal and endothelial cells within the TME, where adenosine signaling upregulates VEGF to drive angiogenesis, reprograms fibroblast metabolism under hypoxia, and alters ECM composition to establish a dense, immune-excluded stroma. These stromal changes parallel the matrix remodeling and fibroblast activation induced by SASP, reinforcing a chronic, immunosuppressive niche that supports tumor survival and therapeutic resistance [74]. Having outlined the functional impact of senescent immune cells on the TME, Section 3 now turns to the upstream factors, genetic, epigenetic and metabolic, that drive immunocyte senescence in ovarian cancer.

## 3 Molecular drivers of immune cell senescence in ovarian cancer

### 3.1 Genetic drivers of ovarian carcinogenesis and their immune consequences

Large-scale sequencing of ovarian epithelial tumors has revealed distinct driver mutations that both initiate malignancy and sculpt the immune microenvironment. BRCA1/2 loss (approximately 20% of high-grade serous) causes HRD, elevating neoantigen burden and activating the cGAS-STING-IFN I axis, which in turn recruits CD8^+^ T cells and DCs and upregulates PD-L1 on tumor and myeloid cells. TP53 mutations (near-universal in serous subtypes) promote chronic NF-κB signaling, favor M2-polarized macrophage infiltration, and increase Treg recruitment via CCL2 and TGF-β secretion. PIK3CA pathway alterations (common in clear-cell/mucinous) drive PI3K/Akt-mediated immunosuppressive cytokine release (IL-10) and exclude effector T cells by modulating the ECM. CCNE1 amplifications (HR-proficient serous) correlate with low neoantigen load, sparse TILs, and an immunologically “cold” phenotype. Integrating these driver-specific programs is critical for understanding heterogeneity in immunotherapy response and for the rational design of combinatorial regimens (Table 2).

**Table 2:** Key genetic drivers of ovarian carcinomas and their associated immune microenvironment features.

Driver mutation	Frequency (%)	Neoantigen load	Key immune features
*BRCA1/2*	15–20	↑↑	CD8^+^ TILs ↑, PD-L1 ↑, and IFN I signaling ↑
*TP53*	∼96	↑	M2 TAMs ↑, Tregs ↑, and NF-κB-driven cytokines
*PIK3CA*	10–15	↔	IL-10 ↑, ECM remodeling, and T-cell exclusion
*CCNE1* amplification	20–25	↓	“Cold” TME and low TIL density

↑↑: significantly increased; ↑: increased; ↔: no change; ↓: decreased. BRCA1/2: breast cancer gene 1/2; TILs: tumor-infiltrating lymphocytes; PD-L1: programmed death-ligand 1; IFN I: interferon type I; TP53: tumor protein p53; M2 TAMs: M2-polarized tumor-associated macrophages; Tregs: regulatory T cells; NF-kB: nuclear factor kappaB; PIK3CA: phosphatidylinositol-4,5-bisphosphate 3-kinase catalytic subunit alpha; IL-10: interleukin-10; ECM: extracellular matrix; CCNE1: cyclin E1; TME: tumor microenvironment.

In addition to oncogenic mutations, immunocyte senescence is further orchestrated by endogenous cellular stressors, telomere attrition, DNA damage response (DDR) activation, and metabolic dysregulation, which are detailed in Section 3.2.

### 3.2 Endogenous factors

#### 3.2.1 Telomere erosion and DDR activation

Telomere erosion and DDR play pivotal roles in immune cell senescence in ovarian cancer, with intricate mechanisms spanning multiple facets. First, telomere shortening emerges as a critical factor. Telomeres, which are repetitive sequences located at the termini of chromosomes, undergo progressive shortening with each cell division. Once telomeres reach a critical short length, they activate the DDR, ultimately leading to cellular senescence or apoptosis [77]. In ovarian cancer, telomere shortening not only impacts cellular proliferative capacity but is also intimately associated with the senescent state of tumor cells [78]. Research has demonstrated that during the senescence of ovarian cancer cells, there is a significant reduction in telomere length, accompanied by decreased activity of the telomerase catalytic subunit, human telomerase reverse transcriptase (hTERT) [78]. Second, DDR activation constitutes a vital aspect of cellular senescence. DDR induces cell cycle arrest by activating signaling pathways such as p53-p21 and p16-p53, thereby preventing uncontrolled cell replication [77]. Additionally, DDR may suppress tumorigenesis and progression by promoting cellular senescence [79]. In ovarian cancer, this reaction is tightly associated with telomere shortening and has been noted in diverse ovarian cancer cell types [80]. Furthermore, the modulation of signaling cascades plays a pivotal role in the process of cellular senescence. For example, the p53-p21 pathway is essential in telomere-induced senescence, where p53 halts cell proliferation by enhancing the expression of p21 [77]. The p16-p53 pathway also contributes to cell senescence induced by telomere shortening. The activation of these signaling pathways not only results in cell cycle arrest but may also induce cell apoptosis or senescence.

#### 3.2.2 Epigenetic regulation: sirtuin 1 (SIRT1) downregulation and histone H3 lysine 9 (H3K9me3) modification

In ovarian cancer, the impact of SIRT1 downregulation and H3K9me3 modification on immune cell senescence can be analyzed from multiple perspectives. SIRT1, an essential deacetylase, participates in diverse cellular processes such as cellular senescence, DNA repair, autophagy, and mitochondrial function [81]. In ovarian cancer, it extends the lifespan of ovarian tissue and regulates ovarian reserve by maintaining chromosomal integrity, regulating adhesion proteins, and participating in primordial follicle activation. Furthermore, SIRT1 inhibits the expression of activation-induced cytidine deaminase (AID), a transcription factor involved in immune responses, by deacetylating H3K9ac and H3K14ac of AID, thereby affecting immune cell function [82].

However, SIRT1 downregulation in ovarian cancer significantly impacts the senescence and function of immune cells. For example, SIRT1 deficiency leads to reduced MHC-II levels in B cells, subsequently affecting the cross-presentation ability of CD4^+^ T cells [82,83], indicating SIRT1's crucial role in regulating immune cell function and antigen presentation. Additionally, SIRT1 downregulation may alter chromatin structure by influencing H3K9me3 modification, thereby affecting gene expression and immune cell senescence. H3K9me3 is a histone modification associated with gene silencing, and SIRT1 inhibits the formation of H3K9me3 by deacetylating H3K9ac, thereby maintaining gene activity [82,84].

Notably, increased H3K9me3 modification is generally associated with gene silencing, potentially affecting the gene expression and function of immune cells. For instance, elevated H3K9me3 may silence certain antigen presentation genes, thereby compromising the capacity of immune cells to detect and eradicate cancer cells [82,85,86]. In ovarian cancer, increased H3K9me3 may further exacerbate immune cell senescence and dysfunction by inhibiting SIRT1 activity [87]. This interaction may promote the progression and dissemination of ovarian cancer.

In summary, SIRT1 downregulation and increased H3K9me3 modification may act synergistically in ovarian cancer, leading to decreased immune cell function and accelerated senescence. This not only impairs the immune cells' ability to combat tumors but may also accelerate the advancement and recurrence of ovarian cancer. Therefore, targeting the regulation of SIRT1 and H3K9me3 may represent a possible treatment approach for ovarian cancer in the future. By restoring SIRT1 activity or reducing H3K9me3 modification, immune cell function can be improved, enhancing their anti-tumor capacity.

#### 3.2.3 Metabolic stress: mitochondrial dysfunction and reactive oxygen species (ROS) accumulation

Mitochondria function as the powerhouses of cells, and when they malfunction, it results in the buildup of ROS, which initiates oxidative stress and subsequently induces cellular senescence [88,89]. Studies have shown that ovarian cancer patients exhibit a high mutation burden in mitochondrial DNA (mtDNA), which may accelerate the senescence process of immune cells [90]. Furthermore, ROS accumulation exacerbates immune cell senescence by activating signaling pathways such as NF-κB [88]. Damage to mtDNA and increased mutation burden are also significant contributors to the aging process of ovarian cancer cells [91]. Oxidative stress plays a pivotal role in causing telomere damage and cellular senescence. Research has shown that it expedites the aging of cells by harming telomeres and DNA [88,92]. Antioxidants such as *N*-acetylcysteine (NAC) can reduce damage caused by oxidative stress, thereby delaying the senescence of ovarian cancer cells [92].

Although endogenous stressors, such as telomere attrition, persistent DNA-damage response activation, mitochondrial dysfunction, and epigenetic drift, can autonomously initiate senescence programs in T cells, NK cells, and other immunocytes, they rarely operate in isolation. Exogenous insults, including genotoxic chemotherapy, radiotherapy, tumor-derived extracellular vesicles carrying senescence-promoting microRNAs, and the chronic inflammatory milieu of the ovarian TME, amplify these intrinsic signals by converging on common downstream effectors (e.g., the p53/p21, NF-κB, and p38 MAPK axes). This convergence enforces irreversible cell-cycle arrest, ROS accumulation, and a mature SASP profile, thereby locking immune cells into a durable senescent state. Acknowledging this synergy between endogenous and exogenous drivers is critical for a comprehensive understanding of immunocyte senescence in ovarian cancer and for the rational design of dual-targeted interventions that both prevent senescence onset and reverse its maintenance.

#### 3.2.4 Emerging mechanisms: epigenetic-metabolic rewiring and therapy-induced senescence

Beyond telomere attrition and the canonical SASP, senescent cells undergo coordinated epigenetic and metabolic reprogramming that reinforces their phenotypic identity and paracrine functions. Dynamic chromatin remodeling, such as loss of repressive H3K9me3 and gain of activating H3K27ac at key enhancer regions, intersects with nutrient-sensing axes (e.g., AMP-activated protein kinase (AMPK) and mTOR) to lock in a pro-SASP transcriptional network [92]. Single-cell RNA sequencing (scRNA-seq) has further revealed transcriptionally and metabolically distinct senescent cell subpopulations: scRNA-seq profiling uncovers clusters with divergent glycolytic versus OXPHOS signatures that correspond to discrete chromatin accessibility states, underscoring pronounced heterogeneity in epigenetic-metabolic rewiring [92]. Importantly, TIS elicits unique rewiring programs: genotoxic treatments generate persistent DNA segments with chromatin alterations reinforcing senescence (DNA-SCARS) and histone depletion, which both amplify cGAS-STING-NF-κB-driven SASP expression and provoke a shift toward fatty acid oxidation, marking a distinct metabolic signature of TIS cells [92]. Finally, integration of single-cell transcriptomics with spatial barcoding has begun to map senescent cell niches *in situ*: combined scRNA-seq and spatial profiling in colorectal cancer delineated SASP-high senescent clusters co-localized with immunosuppressive fibroblast microenvironments, illustrating how spatial multi-omics can resolve cellular heterogeneity and microenvironmental crosstalk [92]. Adapting these approaches to ovarian cancer will be critical for dissecting the epigenetic-metabolic landscape of immunocyte senescence and for identifying spatially restricted therapeutic targets.

### 3.3 Exogenous factors

#### 3.3.1 Chemotherapy-induced premature senescence of immune cells

Chemotherapeutic drugs induce immune cells to enter a senescent state through multiple mechanisms. First, chemotherapy drugs directly kill tumor cells, a process that may prompt the immune system to release more SASP factors, such as IL-6 and CCL2 [93]. Second, chemotherapy drugs not only target tumor cells but also directly induce immune cell senescence, with senescent immune cells secreting more SASP factors, further promoting inflammatory responses and immune suppression in the TME [94]. Additionally, chemotherapy drugs may promote the production of monocytes in the TME by promoting the STAT3 pathway. These monocytes, upon conversion to M1 macrophages, secrete more pro-inflammatory cytokines, exacerbating the inflammatory response in the TME [93].

As chemotherapy progresses, some tumor cells and immune cells may develop drug resistance. These resistant cells continue to secrete SASP factors, maintaining the inflammatory state of the TME, thereby promoting tumor recurrence and metastasis [93]. Simultaneously, senescent immune cells increase PD-L1 expression via the JAK-STAT pathway, activating immune checkpoints, leading to decreased recognition and clearance ability of immune cells towards tumor cells [95]. Additionally, DNA damage and telomerase inactivation induced by chemotherapy drugs are also important mechanisms that induce immune cell senescence, resulting in decreased immune cell function [10]. Furthermore, chemotherapy drugs may further promote immune cell senescence by affecting autophagy and cyclin dysregulation [10].

#### 3.3.2 Tumor-derived exosomes transmitting senescence signals

In ovarian cancer, miR-21 and miR-34a transmitted by tumor-derived exosomes play crucial roles in immune cell senescence and tumor progression. miR-21 affects drug resistance and tumorigenesis in ovarian cancer cells through multiple mechanisms. For example, it induces paclitaxel resistance by binding to the apoptotic protease-activating factor 1 (APAF1) target and exhibits this effect in OVCA432 and SKOV3 cells [96]. Additionally, miR-21 promotes cisplatin resistance by upregulating miR-21-5p in pyruvate dehydrogenase E1 subunit alpha 1 (PDHA1) exosomes [96] and facilitates the occurrence and metastasis of serous ovarian cancer by targeting the tumor suppressor protein PD-4 [97].

MiR-34a induces cellular senescence by downregulating CDK6 [96] and induces drug resistance by targeting the STAT3/caspase (CASP)/ataxia-telangiectasia mutated (ATM) pathway. Exosomal microRNAs (miRNAs) (such as miR-21-3p, miR-433-1p, and miR-985-5p) are abnormally expressed in ovarian cancer cells. These miRNAs not only affect intracellular signaling pathways, promote drug resistance and invasiveness [98], but also influence immune cell function by regulating specific gene expression. For instance, exosomes promote macrophage polarization towards the M2 phenotype, enhancing the immune escape ability of TAMs [99]. These mechanisms collectively drive the progression and development of drug resistance in ovarian cancer.

#### 3.3.3 Chronic inflammatory microenvironment

The chronic inflammatory microenvironment promotes immune cell senescence through the persistent activation of the NF-κB signaling pathway, involving multiple aspects. NF-κB, a crucial transcription factor, promotes the expression of various pro-inflammatory cytokines (such as IL-6 and TNF-α) upon activation [[100], [101], [102]], leading to sustained activation and dysfunction of immune cells in chronic inflammation. Simultaneously, the activation of the NF-κB signaling pathway initiates telomere dysfunction, accelerating telomere shortening and causing premature senescence of immune cells [[103], [104], [105]], further exacerbated by regulating telomerase activity and expression [100].

Moreover, the accumulation of DNA damage in the chronic inflammatory microenvironment is also a significant factor contributing to immune cell senescence. The NF-κB signaling pathway promotes DNA repair by activating the DDR pathway, but when damage cannot be effectively repaired, cells enter a senescent state [100,105]. NF-κB also influences the DNA damage repair process by regulating the activity of tumor suppressor proteins such as p53 [100]. The increase in oxidative stress is another crucial mechanism leading to immune cell senescence in the chronic inflammatory microenvironment. NF-κB reduces oxidative stress-induced damage to cells by activating antioxidant stress pathways (such as nuclear factor erythroid 2-related factor 2 (Nrf2)), but when oxidative stress exceeds the cell's antioxidant capacity, it leads to cellular dysfunction and senescence [100,105].

The persistent activation of the NF-κB signaling pathway also causes immune cells to attain a state where the cell cycle is halted, i.e., senescence, rendering them unable to proliferate and differentiate effectively, thereby resulting in decreased immune function [106]. Furthermore, the activation of inflammasomes (such as NLR family pyrin domain containing 3 (NLRP3)) in the chronic inflammatory microenvironment is also an important mechanism leading to immune cell senescence. The triggering of inflammasomes stimulates the generation of inflammatory cytokines, including IL-1β and IL-18, further exacerbating the inflammatory response and immune cell senescence [101,105].

In summary, the mechanisms by which the chronic inflammatory microenvironment promotes immune cell senescence through the NF-κB signaling pathway involve the expression of pro-inflammatory cytokines, telomere dysfunction, DNA damage and repair, oxidative stress, cell cycle arrest, and the activation of inflammasomes. These mechanisms collectively contribute to the intricate relationship between the persistent inflammatory microenvironment and the senescence of immune cells in ovarian cancer.

## 4 Immune surveillance escape

### 4.1 Hyporesponsiveness of senescent CD8^+^ T cells to tumor antigens

**Fig. 5:**
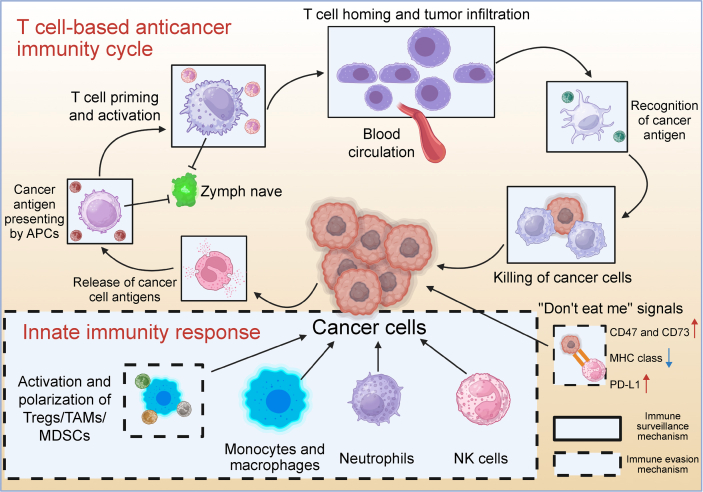
Major mechanisms of cancer immune surveillance and immune escape. Tumor antigens are captured by antigen-presenting cells (APCs) to prime T cells, which traffic to tumors to mediate cytotoxic killing alongside innate immune cells (natural killer (NK) cells and macrophages), while immunosuppressive populations (regulatory T cells (Tregs), tumor-associated macrophages (TAMs), and myeloid-derived suppressor cells (MDSCs)) and tumor immune-evasion strategies (programmed death-ligand 1 (PD-L1), CD47, and major histocompatibility complex (MHC) class I downregulation) counterbalance this response. Therapeutic strategies include enhancing APC priming, improving T cell infiltration, and blocking immunosuppressive signals (CD47 and PD-L1) to optimize immunotherapy efficacy.

With advancing age, immune system function gradually declines, leading to the deterioration of CD8^+^ T cell function. Research has shown that senescent CD8^+^ T cells in ovarian cancer display reduced responsiveness to tumor antigens, which is tightly linked to their impaired antigen recognition and diminished effector capabilities. For instance, senescent T cells may fail to effectively kill tumor cells due to reduced secretion or decreased expression, thereby facilitating tumor escape from immune surveillance [14,107] (Fig. 5). Unlike melanoma or renal cell carcinoma, where PD-1 blockade typically restores CD8^+^ T cell function, ovarian adenocarcinoma TILs embedded in ascites fluid display dampened rescue by checkpoint inhibitors. High lactate and adenosine levels in peritoneal effusions further blunt TCR signaling, and unique CAF-secreted factors (e.g., MFAP5 and tenascin-C) create a physical barrier that deepens T cell metabolic stress. These combined effects generate a refractory exhaustion profile not observed in organ-localized tumors.

In ovarian cancer, the function of CD8^+^ T cells is suppressed by multiple factors. First, although senescent CD8^+^ T cells can rapidly secrete high levels of the effector cytokine IFN-γ upon memory stimulation, their metabolic reprogramming leads to reduced adaptability to glycolysis and OXPHOS, thereby failing to provide sufficient energy to support rapid proliferation and effector functions [108]. Second, ovarian cancer impairs the anti-tumor function of CD8^+^ T cells by inhibiting signaling pathways such as CTLA-4, PD-1, lymphocyte-activation gene 3 (LAG-3), and TIM-3, affecting TCR signaling, reducing cytokine secretion, and leading to T cell dysfunction [108]. Additionally, overexpression of PD-L2 in ovarian cancer is associated with weakened CD8^+^ T cell function and reduced anti-tumor response. As a ligand for PD-1, high PD-L2 expression further inhibits T cell activity [108]. The immune microenvironment of ovarian cancer contributes to CD8^+^ T cell exhaustion by secreting high levels of cytokines (e.g., IL-6 and IL-10) and DC signals. These factors lead to the overactivation of CD8^+^ T cells, ultimately resulting in their exhaustion [109]. Meanwhile, the increased proportion of Treg cells in ovarian cancer further inhibits CD8^+^ T cell activity by secreting inhibitory cytokines (such as IL-10 and TGF-β) and competitively consuming immunoglobulins and nutrients [109]. Moreover, senescent CD8^+^ T cells express markers associated with T cell energy and senescence (such as Killer cell lectin-like receptor subfamily G member 1 (KLRG1), TIM-3, and CD75), indicating their inability to effectively recognize and kill tumor cells [109], thereby rendering them partially dysfunctional in ovarian cancer.

### 4.2 Molecular mechanisms of impaired immunological synapse (IS) formation

The establishment of the IS is a vital structural interaction between T cells and APCs, such as DCs, which is indispensable for T cell activation and the initiation of immune responses. However, with immunosenescence, the expression levels of T cell surface molecules (such as CD8 and CD69) decrease, leading to impaired IS formation and further weakening the killing ability of T cells towards tumor cells [110,111]. Microtubule dynamics play a key role in IS formation, with dynamic changes in tubulin affecting the formation and maintenance of TCR-CD28 microclusters, while abnormal microtubule dynamics may hinder TCR-CD28 microcluster formation, thereby affecting T cell activation [[112], [113], [114]]. Signal transduction and trans-polarization communication play central roles in IS formation, with TCR signal transmission and degradation requiring regulation through multiple signaling pathways. Notably, the regulation of calcium ion concentration and activation of calcium signaling pathways are important components of signal transduction [115].

Signal transduction and trans-polarization communication play central roles in IS formation, with TCR signal transmission and degradation requiring regulation through multiple signaling pathways. Notably, the regulation of calcium ion concentration and activation of calcium signaling pathways are important components of signal transduction [114]. The trans-polarization communication mechanism regulates TCR signals through integrin and other inward and outward signaling mechanisms, maintaining IS stability [116]. Cytoskeletal remodeling is also a significant aspect of IS formation, with actin polymerization and depolymerization affecting IS morphology and function [117], and dynein and Rab6-dependent trafficking influencing endocytic-transcytosis and vesicular transport [117].

Furthermore, various molecular complexes play important roles in IS formation, such as the sorting nexin 27 (SNX27)-retromer complex, Wiskott-Aldrich syndrome protein, and SCAR homolog (WASH) complex, which participate in IS formation and function [118], with the interaction of the SNX27 FERM domain being crucial for IS formation [118]. Other molecular mechanisms, such as the dynamic changes of lymphocyte function-associated antigen 1 (LFA-1) molecules at the intercellular interface [119] and the regulation of calcium ion concentration and activation of calcium signaling pathways [114], also impact IS formation.

### 4.3 Mechanisms of therapeutic resistance

#### 4.3.1 Chemotherapy resistance mediated by senescent T cells

In ovarian cancer patients, senescent T cells promote chemotherapy resistance in tumor cells by activating the IL6/STAT3 signaling pathway. This mechanism may be related to the pro-inflammatory factors (such as IL-6) secreted by senescent T cells, which can induce an immunosuppressive state in the TME, thereby helping tumor cells escape chemotherapy [10,14].

In ovarian cancer, IL-6 is predominantly released by ovarian cancer cells, along with macrophages, fibroblasts, and various other cells within the TME [120]. IL-6 binds to its receptor, activating the JAK/STAT3 signaling pathway, leading to STAT3 phosphorylation and nuclear translocation, and subsequently promoting downstream gene expression [121]. Persistent activation of STAT3 is frequently observed in high-grade epithelial ovarian cancer and is indicative of a poor prognosis [120], as it not only promotes the proliferation and survival of ovarian cancer cells but also enhances tumor cell invasiveness and chemotherapy resistance by promoting EMT [120,122]. IL-6 promotes EMT through STAT3 activation, conferring tumor cells with stronger migration and invasion capabilities and higher resistance to chemotherapy drugs (such as paclitaxel) [120,122]. Additionally, IL-6 inhibits apoptosis by increasing the expression of Bcl-2 family proteins, enhancing the survival ability of ovarian cancer cells [123] and further exacerbating chemotherapy resistance. The IL6/STAT3 signaling cascade also enhances cell survival and proliferation by stimulating the PI3K/Akt/mTOR pathway [124], bypassing the inhibitory effects of chemotherapy drugs and leading to chemotherapy resistance. High levels of IL-6 and high STAT3 expression are closely related to poor prognosis in ovarian cancer patients [120], thus targeting the IL6/STAT3 pathway may be an effective strategy to improve the prognosis of ovarian cancer patients.

#### 4.3.2 SASP promotes CSCs enrichment

Senescent T cells not only affect tumor cells directly but also promote the enrichment and self-renewal of ovarian CSCs by secreting various inflammatory factors (such as IL-6 and IL-8), forming the SASP. This phenotype enhances the invasiveness and therapeutic resistance of tumors [4,10]. On one hand, cytokines (such as IL-6 and IL-8), chemokines, and growth factors in SASP promote the self-renewal and differentiation capabilities of ovarian CSCs. These factors are not only increased in ovarian cancer but are also closely related to cancer metastasis and recurrence [125,126]. For instance, IL-6 can further enrich ovarian CSCs and promote their metastatic ability [127,128], while IL-8 expression is associated with ovarian cancer metastasis, particularly with higher expression levels in the ascites of advanced ovarian cancer patients [129]. On the other hand, SASP promotes the survival and proliferation of ovarian CSCs by altering the TME. The downregulation of ECM and adhesion factors in SASP reduces cell adhesion and increases shedding events, contributing to the survival and diffusion of ovarian CSCs in the TME [127]. Simultaneously, SASP promotes the activation and proliferation of fibroblasts, further remodeling the TME and providing a favorable survival environment for ovarian CSCs [130]. Additionally, SASP enhances drug resistance by reducing the sensitivity of ovarian cancer cells to chemotherapy drugs [131], with the secreted cytokines and growth factors potentially interfering with the action of chemotherapy drugs and protecting ovarian CSCs from drug-induced killing. In summary, SASP promotes the enrichment and self-renewal of ovarian CSCs through multiple mechanisms such as enhancing self-renewal and differentiation capabilities, altering the TME, and increasing drug resistance.

### 4.4 Clinical relevance evidence

#### 4.4.1 Inverse relationship between peripheral blood senescent T cell proportion and patient outcome

Research has demonstrated a strong link between the percentage of senescent T cells in peripheral blood and the prognosis of ovarian cancer patients. An elevated proportion of senescent T cells often signals worse survival rates and a heightened risk of recurrence. This correlation may stem from the diminished functionality of senescent T cells, which impedes the immune system's capacity to eliminate tumors [14,107].

#### 4.4.2 Senescence marker expression in TILs

In patients with ovarian cancer, the levels of senescence markers (including CD57, PD-1, among others) expressed by TILs are elevated. This indicates that TILs undergo immunosenescence within the TME. The heightened expression of these markers correlates with a poor prognosis in ovarian cancer patients, implying that the senescent state of TILs could be a pivotal factor influencing therapeutic outcomes [132,133]. The expression profile of senescence markers in TILs exerts a considerable influence on the therapeutic responses of ovarian cancer patients. Studies have demonstrated that the senescence state of TILs can affect patient prognosis and treatment response through multiple mechanisms. In terms of prognosis, a high proportion of CD3^+^ TILs is linked to survival rates in ovarian cancer patients. Specifically, a higher frequency of CD3^+^ TILs is associated with decreased recurrence rates and extended disease-free survival, suggesting that the presence of these TILs may contribute to suppressing tumor growth and metastasis [134]. Additionally, the presence of TILs is significantly associated with overall survival rates in ovarian cancer patients, with TILs significantly improving survival rates in patients with complete remission who have received platinum-based drugs and radiotherapy [133].

In terms of treatment response, the senescence state of TILs may affect their function. TILs may enhance treatment efficacy by promoting immune clearance in damaged cancer cells [130], but their senescence may also lead to treatment resistance, particularly under altered microenvironmental conditions, such as the influence of TNF. In some cases, the senescence of TILs may limit their anti-tumor effects by reducing inflammatory responses and promoting immune tolerance [130]. Studies have also found that the expression of senescence biomarkers such as senescence-associated beta-galactosidase (SA-β-Gal) and gamma H2A histone family member X (γ-H2AX) in ovarian cancer tissues is associated with patient age, International Federation of Gynecology and Obstetrics (FIGO) stages III-IV, and malignant ascites positivity [135]. SA-β-Gal expression is higher in elderly patients, while γ-H2AX expression is negatively correlated with patient age. The expression levels of these biomarkers can serve as important indicators for assessing the senescence state of TILs, suggesting that the senescence state of TILs may be influenced by patient age and other clinical factors.

## 5 Strategies for targeting immunosenescence in ovarian cancer treatment

### 5.1 Senescent immune cell clearance strategies

Strategies aimed at eliminating senescent immune cells have exhibited considerable promise in the management of ovarian cancer. On one hand, senolytic drugs such as navitoclax and fisetin have proven effective in ovarian cancer models. Navitoclax, as a Bcl-2 inhibitor, enhances immunotherapy efficacy by inducing apoptosis in senescent immune cells, thereby reducing immune suppression and restoring immune cell function. Fisetin, a natural anti-inflammatory compound, eliminates senescent cells by inhibiting the SASP, thus improving immune responses [5,14].

Beyond promising preclinical data, senolytic agents are now advancing into human studies that underscore their translational potential for clearing senescent immune cells. i) Navitoclax (ABT-263): originally developed as a Bcl-2 family inhibitor for hematologic malignancies, navitoclax has been repurposed for senolysis and entered phase I/II clinical trials. In a phase I dose-escalation study combining navitoclax with erlotinib (Trail No.: NCT01009073) [136], patients with advanced solid tumors exhibited a measurable reduction in circulating p16 positive T-cell fractions alongside increased CD8^+^ T-cell cytotoxic markers, without dose-limiting toxicity. Clinical reviews also summarize early-phase idiopathic pulmonary fibrosis trials in which navitoclax monotherapy decreased senescent alveolar epithelial cells and stabilized forced vital capacity over 12 weeks. ii) Fisetin: this natural flavonoid senolytic has progressed into phase I/II human studies. In a randomized trial for knee osteoarthritis (Trial No.: NCT04210986) [137], two monthly cycles of intermittent fisetin (20 mg/kg/day for two days) produced a 25% reduction in Western Ontario and McMaster Universities Osteoarthritis Index (WOMAC) pain scores and a 30% decrease in serum GDF15, a key SASP marker, at 12 weeks. Similarly, a pilot study in post-chemotherapy breast cancer survivors (PROFFi) found that a single three-day course of fisetin reduced circulating IL-6 and IL-8 by 40% and improved frailty index scores at four weeks. These emerging clinical and real-world data demonstrate that senolytic therapies can safely deplete senescent cell populations, attenuate SASP factors, and yield functional benefits in humans. They provide a strong rationale for dedicated ovarian cancer trials to assess optimal dosing, safety, and the impact of senescent immune cell clearance on antitumor immunity.

Alternatively, CAR-T cell therapy, an emerging immunotherapy, targets specific surface markers to eliminate tumor cells. By focusing on senescence-associated surface markers (such as urokinase plasminogen activator receptor (uPAR) and dipeptidyl peptidase-4 (DPP4)), novel CAR-T cell therapies can be developed to more precisely clear rejuvenate senescent immune cells and bolster anti-tumor immune responses [138]. These strategies provide new directions for the treatment of ovarian cancer.

### 5.2 Functional reprogramming of senescent immune cells

Strategies for functional reprogramming of senescent immune cells also exhibit great potential in ovarian cancer treatment. On one hand, a combination therapy of PD-1/CTLA-4 blockade and telomerase activators (such as TERT inhibitors) can prolong the survival of immune effector cells and further enhance immune responses. This combined therapy has shown significant anti-tumor effects in ovarian cancer models [139]. On the other hand, metabolic interventions, such as nicotinamide adenine dinucleotide (NAD) + supplementation or the use of AMPK agonists, can regulate the energy metabolism of immune cells [138,139], thereby enhancing their function and anti-tumor capabilities. These strategies offer new directions for ovarian cancer treatment, potentially improving therapeutic outcomes by reprogramming the function of senescent immune cells.

### 5.3 New paradigms for combination therapies

The combination of chemotherapy and immunotherapy represents an important direction in current ovarian cancer treatment. Research has indicated that chemotherapy can augment the effectiveness of immunotherapy by inducing tumor cell necrosis and modifying the TME. Nonetheless, chemotherapy may also result in immune suppression. Therefore, optimizing the timing of chemotherapy and immunotherapy (e.g., administering chemotherapy first to eliminate some tumor cells, followed by immunotherapy to clear residual tumor cells) may improve therapeutic outcomes [139]. Oncolytic viruses are a type of therapy that can directly kill tumor cells and activate anti-tumor immune responses. Research indicates that oncolytic viruses can induce tumor cells to enter an immunogenic senescence state, thereby enhancing the effectiveness of immune-based treatments. For example, the use of oncolytic viruses in conjunction with immune checkpoint inhibitors (like PD-1/PD-L1 blockers) can markedly enhance anti-tumor outcomes [138,139].

The combination of chemotherapy and immunotherapy represents a promising strategy to overcome the immunosuppressive ovarian cancer microenvironment. Preclinical models have demonstrated clear synergy between cytotoxic agents and immune checkpoint blockade; for example, in the ID8 syngeneic mouse model, anti-PD-1 or PD-L1 antibodies added to paclitaxel significantly prolonged survival and enhanced CD8^+^ T-cell infiltration [138,139]. Building on these findings, several phase III trials are now evaluating chemo-immunotherapy combinations in ovarian cancer:

i) IMagyn050/GOG-3015/ENGOT-OV39 (Trail No.: NCT03038100) [140] is testing the addition of atezolizumab to carboplatin, paclitaxel, and bevacizumab in newly diagnosed stage III/IV patients, with progression-free and overall survival as primary endpoints.
ii) JAVELIN Ovarian 100 (Trail No.: NCT02718417) [141] compared carboplatin/paclitaxel plus avelumab (with subsequent avelumab maintenance) versus chemotherapy alone in treatment-naïve epithelial ovarian cancer, though enrollment was halted for futility.
iii) JAVELIN Ovarian 200 (Trail No.: NCT02580058) [142] evaluated avelumab with pegylated liposomal doxorubicin in platinum-resistant/refractory disease; results to date have been mixed, highlighting the need for better biomarker-driven patient selection.

To provide actionable insight, Table 3 summarizes key preclinical successes alongside the principal ongoing or completed clinical trials of chemo-immunotherapy combinations in ovarian cancer.

**Table 3:** Summary of chemo-immunotherapy combination strategies in ovarian cancer.

Strategy	Preclinical evidence
Anti-PD-1/PD-L1 + paclitaxel	ID8 syngeneic EOC model: improved survival and CD8^+^ T-cell infiltration
Avelumab + carboplatin/paclitaxel	Synergistic cytotoxic effects with checkpoint blockade in preclinical EOC models
Avelumab + pegylated liposomal doxorubicin	Synergistic effects of checkpoint inhibitors with cytotoxic agents in EOC models

PD-1: programmed cell death protein 1; PD-L1: programmed death-ligand; EOC: epithelial ovarian cancer; NCT: National Clinical Trial.

## 6 Challenges and future directions

### 6.1 Challenges in translational medicine

For instance, in a murine S180 sarcoma model, a paradigm of a highly immunosuppressive TME, dense infiltration of CD4^+^CD25^+^Foxp3^+^ Tregs and elevated local levels of IL-10, TGF-β1, and IL-4 foster immune evasion and tumor progression. Direct moxibustion applied to the tumor site markedly reduced both systemic and intratumoral Treg frequencies, downregulated Foxp3, IL-10, and TGF-β1 gene expression within the TME, and concomitantly enhanced effector CD4^+^ and CD8^+^ T cell infiltration and IFN-γ production, collectively shifting the microenvironment toward an antitumor state and inhibiting sarcoma growth [143].

This example underscores the feasibility of modulating immune-stromal interactions by physical or combinatorial approaches, and suggests that analogous strategies, tailored to ovarian cancer's unique immunosenescent landscape, may unlock new avenues for durable TME reprogramming.

#### 6.1.1 Precise identification of tissue-specific senescence markers in ovarian cancer

Identifying tissue-specific senescence markers poses a significant challenge in the treatment and research of ovarian cancer. The heterogeneity of ovarian cancer complicates the identification of molecular markers. For example, studies have found different gene expression patterns in ovarian cancer cell lines and primary cultures, indicating significant heterogeneity in the molecular characteristics of ovarian cancer [144]. Additionally, the immunological milieu of ovarian cancer patients is closely related to metabolic reprogramming and cellular senescence, which may affect therapeutic outcomes [14]. Therefore, further exploration of the molecular mechanisms underlying tissue-specific senescence markers in ovarian cancer, combined with multi-omics data analysis, is required for precise identification.

#### 6.1.2 Selection of therapeutic windows in ovarian cancer: early prevention of senescence vs. clearance of senescent cells

The selection of therapeutic windows in ovarian cancer treatment is another important challenge. Studies have shown that ovarian cancer patients often exhibit no obvious symptoms in the initial phases, resulting in delayed diagnosis and therapeutic intervention [145]. Furthermore, changes in cellular senescence and the immune landscape of ovarian cancer may affect therapeutic outcomes. For instance, metabolic reprogramming of immune cells and the senescent state of tumor cells can influence anti-tumor responses and treatment resistance [14]. Hence, striking a balance between early prevention of cellular senescence and the elimination of senescent cells during therapy represents a crucial avenue for future research endeavors.

### 6.2 Demand for technological innovation

#### 6.2.1 Single-cell multi-omics analysis of heterogeneity in senescent immune cells in ovarian cancer

Single-cell multi-omics technology holds significant value in dissecting the heterogeneity of senescent immune cells in ovarian cancer. For example, scRNA-seq and single-cell proteomics can reveal the molecular characteristics of immune cells in ovarian cancer and their roles in disease progression [146]. Moreover, single-cell analysis offers a means to investigate alterations in the immunological milieu of ovarian cancer patients, thus furnishing a foundation for personalized therapeutic strategies [147].

#### 6.2.2 Ovarian cancer organoid models for simulating immune-tumor interactions

Ovarian cancer organoid models are important tools for studying immune-tumor interactions. By mimicking the immune landscape of ovarian cancer patients, researchers can gain deeper insights into the interplay between immune cells and tumor cells [148]. For instance, in patients with high-grade ovarian cancer, sialic acid-binding immunoglobulin-type lectin 9 (Siglec-9)^+^ TAMs constitute an immunosuppressive population, a phenomenon that can be further investigated using organoid models [148]. Additionally, organoid models can be used to assess the efficacy of immunotherapy, providing guidance for clinical applications [147].

### 6.3 Prospects for clinical research

#### 6.3.1 Personalized senescence-targeted therapy guided by biomarkers in ovarian cancer

Personalized treatment for ovarian cancer requires reliance on precise biomarkers. For example, studies have found that the expression level of the cystatin-S gene (*CST4*) is closely related to the prognosis of ovarian cancer patients [149]. Furthermore, biomarker-based personalized treatment strategies can improve therapeutic outcomes and reduce side effects [150]. Therefore, future research should focus on developing new biomarkers and applying them in clinical practice.

#### 6.3.2 Immunosenescence index as a predictor of therapeutic efficacy in ovarian cancer

The immunosenescence index of ovarian cancer patients may serve as an important indicator for predicting therapeutic efficacy. Research has indicated that immune scores are tightly associated with chemotherapy responsiveness and patient prognosis [144]. Additionally, the immunosenescence index can be used to assess the efficacy of immunotherapy [147]. Therefore, future research should further explore the application value of the immunosenescence index in ovarian cancer treatment.

## 7 Conclusion

In summary, although cellular senescence and immune exhaustion are common across solid tumors, ovarian adenocarcinomas stand out for their ascites-driven SASP amplification and distinct CAF-mediated ECM remodeling. These peritoneal niche-dependent factors synergize to exacerbate CD8^+^ T cell dysfunction and checkpoint inhibitor resistance. Future therapies should therefore combine senomorphic or senolytic agents with strategies that disrupt ascites-specific SASP loops to achieve durable antitumor immunity in ovarian cancer. By regulating the microenvironment of immune cell senescence, it is possible to achieve a paradigm shift in ovarian cancer treatment. Eliminating senescent immune cells and reprogramming their functions can enhance anti-tumor immune responses and overcome treatment resistance. Future research needs to further explore tissue-specific senescence markers and the selection of therapeutic windows, combined with single-cell multi-omics technology and organoid models to develop personalized treatment regimens and improve the prognosis of ovarian cancer patients.

## CRediT authorship contribution statement

**Xiang Li:** Writing – original draft, Formal analysis, Data curation. **Xian Li:** Writing – original draft, Resources, Methodology, Investigation. **Sha Ni:** Writing – review & editing, Supervision. **Xiaohui Zhang:** Validation, Resources, Methodology. **Bingnan Liu:** Writing – review & editing, Project administration, Funding acquisition, Conceptualization.

## Declaration of competing interest

The authors declare that they have no known competing financial interests or personal relationships that could have appeared to influence the work reported in this paper.
